# Effects of positive psychological intervention on psychosocial adjustment in childbearing-age patients with breast cancer

**DOI:** 10.3389/fonc.2026.1780992

**Published:** 2026-05-08

**Authors:** Hongtao Chen, Shijin Xu, Ping Hu, Yuanyuan Liu, Lihua Wen, Xiaohua Song, Shili Luo

**Affiliations:** 1 School of Nursing, Hunan University of Chinese Medicine, Changsha, Hunan, China; 2 The Second Hospital of Integrated Traditional Chinese and Western Medicine, Hunan University of Chinese Medicine, Liuyang, Hunan, China; 3 Yiyang Central Hospital/Yiyang Central Hospital Affiliated to Hunan University of Chinese Medicine, Yiyang, Hunan, China; 4 The Affiliated Cancer Hospital of Xiangya School of Medicine, Central South University/Hunan Cancer Hospital, Changsha, Hunan, China

**Keywords:** breast cancer of childbearing age, positive psychology, post-traumatic growth, self-concept, social support

## Abstract

**Objective:**

This study mainly explores the effect of positive psychological intervention on the psychosocial adjustment of breast cancer patients of childbearing age.

**Methods:**

A total of 130 childbearing-age breast cancer patients admitted to our hospital were enrolled as research subjects and randomly divided into a control group and an intervention group, with 65 cases in each group. The control group received routine nursing care for breast cancer, while the intervention group was given a positive psychological intervention program based on the PERMA model on the basis of the same routine nursing care. The Psychosocial Adjustment to Illness Scale-Self Report (PAIS-SR) was used to assess the primary outcome of psychosocial adjustment, and the Tennessee Self-Concept Scale (TSCS), Interpersonal Relationship Comprehensive Diagnostic Scale (IRIDS), Social Support Scale (SSS) and Posttraumatic Growth Scale (PTG) were applied to evaluate self-concept, interpersonal relationship, social support and posttraumatic growth, respectively. All above assessments were conducted at baseline, immediately after the intervention and at the 6-month follow-up.

**Results:**

Childbearing-age breast cancer patients exhibited low psychosocial adjustment. Following positive psychological intervention, the intervention group demonstrated significantly higher scores in all dimensions of the TSCS and SSS, and significantly lower scores in all dimensions of the IRIDS (*P* < 0.05). Furthermore, the intervention group scored significantly higher than the control group in all dimensions of the PTG (*P* < 0.05). A positive correlation was observed between the total SSS score and the total PTG score (r = 0.635, *P* < 0.05).

**Conclusion:**

Positive psychological intervention significantly enhances self-concept, improves interpersonal relationships, strengthens social support, and promotes post-traumatic growth in childbearing-age breast cancer patients, ultimately elevating their level of psychosocial adjustment and enhancing their quality of life.

## 1 Introduction

Breast cancer is a serious and potentially life-threatening disease that imposes substantial physical and psychological burdens on women. Its incidence has been steadily increasing worldwide, with a noticeable trend toward younger age at onset (1–3). Early-stage breast cancer patients can achieve a high cure rate through mastectomy, radiotherapy, and chemotherapy (4, 5). At the same time, the loss of breasts will damage their image and cause inferiority complex, which will also affect the intimate relationship between husband and wife and cause family crisis (6, 7). The curious or judgmental gazes of friends and relatives can make individuals feel discriminated against, leading to social withdrawal, negative psychological states, and difficulties in psychosocial adjustment (8, 9). Psychosocial adjustment refers to patients’ self-evaluation, cognitive attitude, emotional response, and coordination with family, group, and society when they are stressed (10). The psychosocial adjustment of breast cancer patients seriously affects their quality of life and prognosis improvement (11, 12). Therefore, the psychosocial adjustment of breast cancer patients of childbearing age warrants greater attention.

In recent years, the increasing application of positive psychology research has demonstrated the effectiveness of positive psychological interventions in improving personal meaning, mental health, and adaptation to cancer (13, 14). Traditional psychology primarily focuses on patients’ negative mental states and resolving psychological problems. Positive psychology, in contrast, shifts the focus to patients’ positive psychological qualities and the mobilization of positive emotions, representing a transformation from the traditional approach (14). Following the application of positive psychology interventions, it has been observed that patients, while exhibiting negative psychological states, also demonstrate positive psychological characteristics such as post-traumatic growth (15, 16). Encouraging patients to focus on their positive psychological traits helps distressed breast cancer patients adjust psychosocially. Actively coping with treatment-related challenges and pressures is critical for promoting psychosocial adjustment, reducing the burden of disease, and improving quality of life (17–19). This study, therefore, focuses on exploring the influence of positive psychological interventions on the psychosocial adjustment of childbearing-age breast cancer patients following cancer trauma. The aim is to provide a theoretical basis for psychosocial rehabilitation strategies tailored to this population.

## 2 Materials and methods

### 2.1 Subject investigated, object of study

Using a convenience sampling method, breast cancer patients were selected as the study subjects from the Department of Breast and Thyroid Surgery at a Grade A tertiary hospital in Hunan Province from January 2024 to December 2024.

The inclusion criteria were: (1) pathological diagnosis of breast cancer; (2) age between 20 and 45 years; (3) ability to understand the questionnaire and provide accurate responses; (4) expected survival time longer than 12 months; and (5) voluntary participation with written informed consent. The exclusion criteria included: (1) severe complications or diagnosed psychiatric disorders; (2) impaired comprehension ability; (3) non-primary breast cancer; (4) history of other malignant tumors; and (5) participation in other psychological intervention studies.

Sample size was calculated using G*Power 3.1 software based on the primary outcome of psychosocial adjustment, which was measured by the Psychosocial Adjustment to Illness Scale-Self Report (PAIS-SR).

Assuming a medium effect size (Cohen’s d = 0.5) based on previous studies of positive psychological interventions in breast cancer populations, a two-tailed significance level of α = 0.05, and a statistical power of 1−β = 0.80, the minimum required sample size was estimated to be 57 participants per group. Considering a potential attrition rate of 10–20%, commonly observed in longitudinal studies involving cancer patients, the final sample size was increased to 65 participants per group, resulting in a total sample of 130 participants. A random allocation sequence was generated using a random number table method with SPSS 21.0 software. The allocation scheme was placed into sequentially numbered, sealed, opaque envelopes by a third party not involved in the study. Upon enrollment, participants were assigned to groups as the envelopes were opened in sequential order according to the scheme inside.

During the study period, three participants withdrew from the trial, resulting in a final follow-up rate of 97.6%. Specifically, two participants in the control group discontinued the study (one due to personal reasons and the other due to loss to follow-up), while one participant in the intervention group withdrew due to relocation.

All statistical analyses were performed according to the intention-to-treat (ITT) principle, and included all 130 randomized participants. Missing data were handled using multiple imputation (MI). Five imputed datasets were generated based on baseline characteristics and available outcome measurements. To minimize bias caused by missing data and to preserve the integrity of the ITT population, the final results were obtained by pooling estimates across the imputed datasets according to Rubin’s rules.

### 2.2 Intervention methods

The control group adopted routine nursing methods, including: (1) introduction to breast cancer, (2) breast cancer treatment, (3) medication guidance, (4) common side effects and precautions of chemotherapy, and (5) dietary guidance. The intervention group received positive psychological intervention based on routine nursing. The positive psychological intervention program was developed based on Seligman’s PERMA model, which constitutes the five core elements of well-being: Positive Emotion, Engagement, Relationships, Meaning, and Accomplishment.

(20). This theoretical framework guided the design of the nine weekly themes to systematically enhance participants’ psychosocial well-being.

#### 2.2.1 Intervention methods in the intervention group

##### 2.2.1.1 Create a research team

An intervention team composed of 1 attending breast surgeon, 1 rehabilitation doctor, 1 nationally certified psychological consultant, and 3 senior breast surgery nurses, led by the department’s head nurse.

The attending surgeon provides medical authority and expertise, offering specialized guidance on surgical outcomes, aesthetic concerns, and reproductive health issues related to breast cancer treatment. The psychological consultant leads intervention sessions that focus on enhancing psychological well-being, facilitating emotional processing, and supporting meaning-making in the context of illness. The rehabilitation doctor advises on appropriate activity engagement and strategies to support physical recovery, tailored to the individual’s treatment phase and functional status. The senior nurses co-facilitate most sessions, ensure continuity of care, deliver individualized emotional and practical support, and provide hands-on nursing guidance throughout the intervention period.

##### 2.2.1.2 Develop an intervention plan

Formulation of intervention plan: Referring to relevant literature on topics such as chemotherapy, positive psychology, and breast cancer in women of childbearing age, we established a research group focused on positive psychological intervention for this population. We then formulated a first draft of a pre-test intervention plan and collected pre- and post-test feedback.

##### 2.2.1.3 Regular quality control

We held weekly meetings to discuss quality control results. All staff members used brainstorming to analyze existing and potential problems and to formulate predictive treatment and nursing plans. Specific measures were adjusted as needed based on past results. Before implementing subsequent intervention measures, medical staff were trained to ensure research quality and improve effectiveness.

##### 2.2.1.4 Intervention implementation

Interventions were conducted weekly in 40–50 minute sessions, for a total of 9 weeks. See **Table 1** for intervention content. Prior to implementation, the intervention protocol was refined via a two-round Delphi process with 10 experts in breast surgery, oncology nursing, psychology, and reproductive medicine. After evaluating the importance and feasibility of each component, they achieved a high expert authority coefficient (0.88) and significant consensus (Kendall’s W = 0.35 for importance, p < 0.01; W = 0.29 for feasibility, p < 0.01).

**Table 1:** Positive psychological intervention method.

Intervention time	Theme of item	Concrete intervention measures	Intervention lead
First time	Disease and positive cognition of self	a. Actively communicate with patients to understand their psychological characteristics. b. Encourage patients to express their true thoughts and face up to diseases and psychological problems. c. Provide an explanation of breast cancer to give patients a basic understanding of the disease.	Psychological consultant + Senior nurse
Second time	Individual aesthetic guidance	a. In the process of treatment and communication, nurses collect patients’ personal information and give interesting aesthetic guidance. b. Reassure patients that the minimal surgical incision will have a negligible impact on their feminine contours. Breast reconstruction can restore three-dimensional volume and symmetry, enhancing self-esteem through techniques such as breast reconstruction, pocket modification, and the subsequent use of breast implants.	Attending surgeon + Senior nurse
Third time	Reproductive safety information and reproductive hope	a. Combined with the pathological classification and treatment plan of breast cancer patients of childbearing age, the ideal time and age for conception and childbirth were explained. b. Inform patients that assisted reproductive technologies include oocyte cryopreservation, embryo cryopreservation, ovarian tissue cryopreservation, ovarian tissue transplantation, and other similar techniques. c. Share the experiences and emotional journeys of breast cancer patients who have successfully given birth. d. Explain the safety data regarding the absence of recurrence in breast cancer patients who have become pregnant, both domestically and internationally, as well as the data on newborns without malformations.	Attending surgeon
Fourth time	Family support	a. Spouse support: Offer personalized guidance tailored to specific situations. Encourage a positive mindset and address any misconceptions. Help patients strengthen their intimate relationship and fulfill their needs for love and belonging. b. Support from relatives and friends: Help patients recognize and effectively utilize the support available from their network of relatives and friends.	Senior nurse + Psychological consultant
Fifth time	Positive emotion	a. Conduct interviews around the concept and function of positive emotions. b. Cultivate patients’ positive thinking and improve their positive attitude towards events. c. Encourage them to use positive language to improve their mood. d. Praise patients in times when they show positive emotions.	Psychological consultant
Sixth time	Engagement	a. Discuss the relevant topics of investment with patients, and carry out activities according to patients’ condition and their hobbies. b. Encourage patients to plan their goals at this stage according to their physical condition, so that patients can experience the state of devotion more, thus shifting their attention from illness to other places and reducing negative emotions.	Rehabilitation doctor + Senior nurse
Seventh time	Positive interpersonal relationship	a. Conduct interviews with patients around interpersonal relationships. b. Introduce the skills and methods of establishing positive interpersonal and communication with patients. c. Encourage patients to actively participate in social activities to make friends and expand their social circles. Consider organizing patient-to-patient exchange meetings where they can share difficult emotions and coping strategies. Additionally, suggest joining postoperative rehabilitation associations.	Psychological consultant + Senior nurse
Eighth time	meaning	a. Interview with patients about the meaning and value of life. b. Explain that everyone is a unique individual in the world. c. Share success stories to inspire patients to maintain a positive outlook and cope with challenges.	Psychological consultant
Ninth time	achievement	a. Conduct interviews with patients around achievements to explain the positive psychological effects of a sense of accomplishment. b. Instruct patients to establish attainable short-term goals, aligned with their individual strengths and capabilities, within the context of disease management and daily living, and to actively pursue these goals.	Psychological consultant + Senior nurse

### 2.3 Observation index

#### 2.3.1 Psychosocial adjustment to illness scale-self report

The Psychosocial Adjustment to Illness Scale-Self Report (PAIS-SR), developed by L. R. Derogatis (1986), was used to fully evaluate psychosocial adjustment to illness (21). The scale comprises 46 items grouped into 7 domains: Healthcare Orientation (6 items), Vocational Environment (6 items), Domestic Environment (7 items), Social Environment (6 items), Sexual Relationships (5 items), Psychological Distress (9 items), and Extended Family Relationships (7 items). Each item is rated on a 4-point Likert-type scale, from 0 (Not at all) to 3 (Extremely). Total scores range from 0 to 138, with lower scores reflecting better psychosocial adjustment. In this study, the Chinese version of the scale demonstrated good internal consistency (Cronbach’s α = 0.85) and has been widely validated in oncological populations.

#### 2.3.2 Tennessee self-concept scale

The Tennessee Self-Concept Scale (TSCS), originally developed by Fitts (1965), was used to evaluate self-concept (22). The scale comprises 45 items, categorized into three primary dimensions: Structural Dimension (Self-perception, Self-satisfaction, and Self-behavior), Content Dimension (Physical Self, Moral Self, Psychological Self, Family Self, and Social Self), and Evaluative Dimension (Self-criticism). Each subscale includes 5 items. Responses are recorded using a 5-point Likert scale, ranging from 1 (Completely disagree) to 5 (Completely agree). Elevated scores on the Structural and Content dimensions indicate a more positive self-concept, while lower scores on the Evaluative Dimension suggest greater self-acceptance. The Chinese adaptation of the TSCS showed acceptable reliability within the current sample (Cronbach’s α = 0.82).

#### 2.3.3 Interpersonal relationship comprehensive diagnostic scale

The Interpersonal Relationship Comprehensive Diagnostic Scale (IRIDS) was developed by Garthoeffner J. L. (1993) to assess difficulties in interpersonal functioning (23). The instrument comprises 28 dichotomous items (Yes/No) spanning four dimensions: Conversation, Sociality and Friendship, Interpersonal Interaction, and Heterosexual Communication. Items are scored 1 for Yes and 0 for No. Total scores range from 0 to 28, with elevated scores indicative of greater interpersonal difficulties. Interpretive cut-offs are as follows: 0–8 (mild), 9–14 (moderate), 15–20 (severe), and >20 (very severe). In the present study, the scale demonstrated good internal consistency (Cronbach’s α = 0.81).

#### 2.3.4 Social support scale

The Social Support Rating Scale (SSRS), developed by Xiao S. Y. (1994), was used to measure perceived social support (24). The scale comprises 10 items, categorized into three dimensions: Objective Support (3 items), Subjective Support (4 items), and Support Utilization (3 items). Items are rated on a Likert scale ranging from 1 to 5, with total scores ranging from 10 to 40. Higher scores indicate greater perceived social support. The scale has demonstrated good reliability and validity in Chinese populations. In this study, Cronbach’s α was 0.79.

#### 2.3.5 Posttraumatic growth

Posttraumatic growth was measured using the Chinese version of the Posttraumatic Growth (PTG), originally developed by Tedeschi & Calhoun (1996) (25). The inventory includes 21 items across five dimensions: Relating to Others (7 items), New Possibilities (5 items), Personal Strength (4 items), Spiritual Change (2 items), and Appreciation of Life (3 items). Each item is rated on a 6-point scale from 0 (I did not experience this change) to 5 (I experienced this change to a very great degree). Total scores range from 0 to 105, with higher scores indicating greater posttraumatic growth. The Chinese version has demonstrated good psychometric properties, and in this study, Cronbach’s α was 0.85.

### 2.4 Statistical methods

Statistical analyses were performed using SPSS version 21. Continuous variables were expressed as mean ± standard deviation. Baseline comparisons were performed using independent-samples t-tests. Changes over time between groups were analyzed using repeated-measures analysis of variance. A two-sided P < 0.05 was considered statistically significant.

## 3 Result

### 3.1 Comparison of baseline data between the two groups

Baseline demographic and clinical characteristics were collected and are presented in **Table 2**. No statistically significant differences were observed between the two groups with respect to age, disease duration, or other baseline variables (P > 0.05), indicating adequate comparability between groups.

**Table 2:** Comparison of basic data between two groups.

Item	Control group (n=65)	Intervention group (n=65)	χ^2^/*t* value	*P* value
Age (years)	35.03 ± 5.32	35.89 ± 5.40	0.915	0.362
Degree of education (n)			0.039	0.843
Below junior middle school	17	18		
Above junior middle school	48	47		
Duration of the disease (years)	3.83 ± 0.24	3.77 ± 0.96	0.489	0.626
BMI (kg/m^2^)	23.35 ± 1.64	23.53 ± 1.56	0.641	0.523
Neoplasm staging (n)			0.132	0.716
IˎII stage	42	40		
IIIˎIV stage	23	25		
Marriage status (n)			0.381	0.537
Married	51	48		
Divorced, widowed, or single	14	17		

### 3.2 Comparison of PAIS-SR and TSCS scores between the two groups at baseline and after intervention

**Table 3** shows that the two groups had no significant difference in their PAIS-SR total scores before the intervention (P > 0.05). Following the intervention, the intervention group’s total PAIS-SR scores were significantly lower than both the control group’s scores and their own baseline scores at the 6-month follow-up (P < 0.05).

**Table 3:** Comparison of PAIS-SR total score between the two groups before and after intervention (Mean ± SD).

Time	Control group	Intervention group	t	*P* value
Pre	55.42 ± 6.33	56.12 ± 5.86	0.654	0.514
Post	48.49 ± 4.92^*^	42.34 ± 4.83^*^	7.192	<0.01
Post 6 month	45.59 ± 4.87^*#^	38.08 ± 4.13^*#^	9.482	<0.01

^*^ Comparisons with intra-group Pre were statistically significant. ^#^Comparisons with intra-group Post were statistically significant. *P* < 0.05.

Prior to the intervention, the two groups showed no significant differences in scores for the structural dimension, content dimension, or self-criticism on the TSCS (*P* > 0.05). Following the intervention, and at the 6-month follow-up, the intervention group scored significantly higher on the structural and content dimensions, and significantly lower on self-criticism, compared to the control group (*P* < 0.05; see **Table 4**).

**Table 4:** Comparison of TSCS score between the two groups before and after intervention (Mean ± SD).

Time	Control group	Intervention group	*t*	*P* value
Structural dimension				
Pre	45.39 ± 3.43	44.97 ± 3.53	0.688	0.493
Post	51.95 ± 2.34^*^	56.77 ± 2.24^*^	12.000	<0.01
Post 6 month	64.94 ± 4.09^*#^	70.77 ± 3.88^*#^	8.337	<0.01
Content dimension				
Pre	52.15 ± 3.52	51.51 ± 3.62	1.022	0.309
Post	61.72 ± 4.37^*^	64.67 ± 3.99^*^	4.019	<0.01
Post 6 month	67.63 ± 5.29^*#^	72.06 ± 6.02^*#^	4.457	<0.01
Self-criticism				
Pre	17.48 ± 1.39	17.26 ± 1.50	0.867	0.387
Post	16.22 ± 5.12	12.31 ± 3.01^*^	5.308	<0.01
Post 6 month	13.19 ± 2.23^*#^	9.63 ± 2.09^*#^	9.391	<0.01

*Comparisons with intra-group Pre were statistically significant. #Comparisons with intra-group Post were statistically significant. *P* < 0.05.

### 3.3 Comparison of IRIDS score between the two groups before and after intervention

Prior to the intervention, no significant differences were observed between the two groups in any dimension of the IRIDS (Conversation, Communication and Friendship, Social Interaction, Interaction with the Opposite Sex) or in the total score (*P* > 0.05). Following the intervention, and at the 6-month follow-up, the intervention group exhibited significantly lower scores in all IRIDS dimensions and the total score compared to both the control group and their own pre-intervention scores (*P* < 0.05), as detailed in **Table 5**.

**Table 5:** Comparison of IRIDS score between the two groups before and after intervention (Mean ± SD).

Time	Control group	Intervention group	t	*P* value
Conversation				
Pre	5.22 ± 0.51	5.25 ± 0.50	0.339	0.735
Post	4.55 ± 0.50	3.92 ± 0.27^*^	8.938	<0.01
Post 6 month	3.66 ± 0.47^*#^	3.06 ± 0.24^*#^	9.166	<0.01
Communication and friendship				
Pre	5.86 ± 0.52	5.77 ± 0.42	1.086	0.280
Post	4.66 ± 0.47	4.11 ± 0.31^*^	7.876	<0.01
Post 6 month	3.75 ± 0.47^*#^	3.12 ± 0.33^*#^	8.844	<0.01
Social interaction				
Pre	6.15 ± 0.40	6.19 ± 0.39	0.577	0.565
Post	5.39 ± 0.49	4.74 ± 0.44^*^	7.957	<0.01
Post 6 month	4.91 ± 0.29^*#^	3.95 ± 0.21^*#^	21.621	<0.01
Interaction with opposite sex				
Pre	4.75 ± 0.50	4.82 ± 0.49	0.806	0.422
Post	3.92 ± 0.27	3.02 ± 0.27^*^	19.001	<0.01
Post 6 month	3.01 ± 0.27^*^	2.82 ± 0.39^*#^	3.229	<0.01
Total score				
Pre	21.97 ± 1.10	22.02 ± 0.90	0.284	0.777
Post	18.54 ± 1.07^*^	15.79 ± 0.69^*^	17.412	<0.01
Post 6 month	15.34 ± 0.86^*#^	12.95 ± 0.57^*#^	18.684	<0.01

*Comparisons with intra-group Pre were statistically significant. #Comparisons with intra-group Post were statistically significant. *P* < 0.05.

### 3.4 Comparison of SSS score between the two groups before and after intervention

At baseline, there were no significant differences between the intervention and control groups in objective support, subjective support, support utilization, or total SSS score (*P* > 0.05). Following the intervention, and at the 6-month follow-up, the intervention group demonstrated significantly higher scores in all SSS dimensions and total SSS score compared to both the control group and their own baseline scores (*P* < 0.05; see **Table 6**).

**Table 6:** Comparison of SSS score between the two groups before and after intervention (Mean ± SD).

Time	Control group	Intervention group	t	*P* value
Objective support				
Pre	6.45 ± 0.50	6.42 ± 0.52	0.335	0.738
Post	9.60 ± 0.97^*^	11.09 ± 1.55^*^	6.570	<0.01
Post 6 month	10.92 ± 1.53^*#^	12.11 ± 1.52^*#^	4.449	<0.01
Subjective support				
Pre	10.12 ± 1.85	9.89 ± 1.39	0.801	0.424
Post	10.99 ± 1.12	12.34 ± 1.88^*^	4.974	<0.01
Post 6 month	11.92 ± 2.06^*^	13.37 ± 1.53^*#^	4.556	<0.01
Utilization of support				
Pre	7.26 ± 0.53	7.23 ± 0.46	0.345	0.731
Post	9.51 ± 1.60^*^	10.26 ± 1.82^*^	2.495	<0.05
Post 6 month	10.23 ± 1.23^*^	11.32 ± 2.54^*#^	3.114	<0.01
Total score				
Pre	23.83 ± 2.15	23.54 ± 1.67	0.859	0.392
Post	30.09 ± 2.13^*^	33.69 ± 3.00^*^	7.889	<0.01
Post 6 month	33.08 ± 2.87^*#^	36.80 ± 2.28^*#^	8.182	<0.01

*Comparisons with intra-group Pre were statistically significant. #Comparisons with intra-group Post were statistically significant. *P* < 0.05.

### 3.5 Comparison of PTG score between the two groups before and after intervention

As demonstrated in **Table 7**, routine nursing was associated with an increase in the total score of PTG and all dimensional scores, although this increase was not statistically significant (*P* > 0.05). Prior to positive psychological intervention, there were no significant differences in any dimension of post-traumatic growth between the two groups (*P* > 0.05). Following positive psychological intervention and at the 6-month follow-up, the intervention group exhibited significantly higher scores than the control group in the five dimensions of relationship to others, new possibilities, personal strength, spiritual change, and appreciation of life (*P* < 0.01).

**Table 7:** Comparison of PTG score between the two groups before and after intervention (Mean ± SD).

Time	Control group	Intervention group	t	*P* value
Relating to others				
Pre	17.59 ± 4.30	17.46 ± 3.13	0.197	0.844
Post	18.39 ± 4.41	20.95 ± 3.88^*^	3.514	< 0.01
Post 6 month	21.40 ± 4.81^*#^	24.68 ± 4.91^*#^	3.847	< 0.01
New possibilities				
Pre	8.11 ± 2.08	8.09 ± 2.13	0.054	0.957
Post	8.83 ± 2.00	10.03 ± 2.81^*^	2.805	< 0.01
Post 6 month	10.54 ± 2.42^*#^	13.11 ± 2.00^*#^	6.600	< 0.01
Personal strength				
Pre	11.75 ± 3.34	11.89 ± 3.48	0.234	0.815
Post	12.03 ± 2.69	13.68 ± 2.86^*^	3.388	< 0.01
Post 6 month	14.02 ± 3.21^*#^	15.25 ± 3.20^*#^	2.184	< 0.05
Spiritual change				
Pre	4.72 ± 0.45	4.65 ± 0.48	0.858	0.393
Post	4.78 ± 0.41	5.17 ± 0.38^*^	5.625	< 0.01
Post 6 month	4.95 ± 0.48^*^	6.85 ± 0.44^*#^	23.520	< 0.01
Appreciation of life				
Pre	9.03 ± 0.74	9.25 ± 0.56	1.911	0.058
Post	10.59 ± 1.09	11.75 ± 1.08	2.507	< 0.05
Post 6 month	11.68 ± 0.56^*#^	13.22 ± 1.72^*#^	7.011	< 0.01
Total score				
Pre	51.20 ± 5.62	51.34 ± 4.88	0.033	0.974
Post	54.62 ± 5.52^*^	61.59 ± 5.87^*^	6.974	< 0.01
Post 6 month	62.58 ± 6.22^*#^	73.09 ± 7.05^*#^	9.013	< 0.01

*The comparisons of indexes within-group was statistically significant. *P* < 0.05; ^#^Comparisons with intra-group Post were statistically significant. *P* < 0.05.

### 3.6 Pearson correlation analysis between SSS and PTG score

Pearson correlation analysis revealed a positive correlation between the total PTG score and the total SSS score (r = 0.635, *P* < 0.05). With the exception of the “Spiritual change” dimension of PTG and the “Objective support” dimension of SSS (*P* > 0.05), all other dimensions exhibited significant positive correlations (*P* < 0.05), as detailed in **Table 8**.

**Table 8:** Pearson correlation analysis between SSS and PTG score.

Item	Objective support	Subjective support	Utilization of support	SSS total score
Relating to others	0.372^*^	0.526^*^	0.368^*^	0.523^*^
New possibilities	0.286^*^	0.468^*^	0.435^*^	0.478^*^
Personal strength	0.228^*^	0.384^*^	0.479^*^	0.512^*^
Spiritual change	0.175^#^	0.273^*^	0.241^*^	0.286^*^
Appreciation of life	0.339^*^	0.562^*^	0.347^*^	0.557^*^
PTG Total score	0.417^*^	0.613^*^	0.532^*^	0.635^*^

^*^ *P* < 0.05; ^#^*P* > 0.05.

## 4 Discussion

Breast cancer is increasingly understood through an integrative biopsychosocial model that considers psychological, behavioral, and social factors (26, 27). Breast cancer patients of childbearing age frequently experience significant psychological distress stemming from alterations in self-perception and diminished self-esteem, potentially resulting in impaired psychosocial adjustment. Conversely, individuals exhibiting strong psychological and social adaptability tend to adopt a more positive outlook in managing their condition, effectively leveraging self-efficacy and demonstrating a healthy understanding and acceptance of themselves (28, 29). Therefore, identifying an appropriate nursing intervention model is critical to ensuring patients’ quality of life, reducing negative emotions, and improving psychosocial adjustment. Self-concept, interpersonal relationships, social support, and post-traumatic growth are key factors influencing psychosocial adjustment. This study explores the influence of positive psychological intervention on these factors, aiming to provide a theoretical basis for the psychosocial rehabilitation of childbearing-age breast cancer patients.

This study revealed that the psychosocial adjustment level of childbearing-age breast cancer patients was low, potentially due to the various side effects experienced during diagnosis and treatment (30, 31). However, after routine nursing, both their psychosocial adjustment and quality of life significantly improved. This may be attributed to the high proportion of survivors in this study with a disease course of about 3 years; patients with longer survival periods tend to be more optimistic when combined with routine nursing. Relevant studies have shown that the physical function of breast cancer patients gradually recovers 12 months after diagnosis, and the negative emotions caused by the disease diagnosis are significantly alleviated (32). A low level of psychosocial adjustment in childbearing-age breast cancer patients is associated with a reduced quality of life, particularly before routine nursing interventions (17) This suggests that medical staff should clearly recognize that breast cancer is not only a physical disease but also a social and psychological one. While controlling disease progression, they should actively attend to patients’ psychosocial adjustment to improve their quality of life.

To address this deficiency, we implemented positive psychological interventions for breast cancer patients of childbearing age, focusing on self-image, family crisis, and fertility concerns. Post-intervention assessment using the PAIS-SR revealed a significant decrease in patients’ total scores, shifting from a higher range to a moderate range. Notably, improvements were observed across three of the seven dimensions: healthcare orientation (e.g., increased treatment adherence), psychological distress (e.g., reduced anxiety and depressive symptoms), and social environment (e.g., greater participation in social activities). These findings suggest that the positive psychological intervention effectively enhanced patients’ cognitive management of disease-related issues and emotional regulation, thereby facilitating improved integration into social roles and daily life. Regarding self-perception, higher TSCS scores in the intervention group may be attributed to targeted aesthetic guidance (e.g., provision of breast reconstruction information) and the cultivation of positive cognition, which aided in rebuilding confidence following physical changes. In terms of social functioning, lower IRIDS scores reflected improved interpersonal function, likely resulting from communication skills training and spousal support guidance. Reducing interpersonal distress contributes to alleviating isolation, a key factor in psychosocial adjustment (17, 33).

The PTG of patients was comprehensively assessed. Results indicated that while patients receiving routine care exhibited an increase in PTG scores and demonstrated improved psychosocial adjustment at follow-up compared to immediate post-intervention assessment, their overall scores remained relatively low. This suggests that routine care, while potentially effective in alleviating negative emotions, primarily focuses on physiological management, with limited attention to psychosocial dimensions such as emotional support, self-growth, and meaning-making (34). The intervention group showed higher SSS scores, indicating that positive psychological intervention effectively mobilized both objective support (e.g., medical resources) and subjective support (e.g., family care). Such social support can buffer the negative impact of illness-related stress (35). The intervention group reported higher PTG scores, which correlated with their levels of social support. This suggests that positive emotions and social connections can facilitate growth following adversity. Therefore, interventions aimed at enhancing social support may effectively promote positive growth among individuals affected by trauma. Specifically, sharing positive childbirth experiences may reinforce a sense of new possibilities, which could explain the strong correlation observed between PTG and perceived social support (36, 37).

To sum up, the significant changes in the scores of various indicators after the positive psychology intervention suggest that positive psychology can better adjust patients’ social function and emotional state, improve their level of psychosocial adjustment, and thus improve their quality of life. This study developed a positive psychological intervention program for breast cancer patients of childbearing age, verified its applicability in the psychosocial rehabilitation of this patient population, and provides theoretical references for connecting theoretical frameworks and practical programs in the field of psychological intervention for breast cancer patients of childbearing age. However, our research still has some limitations. For example, because we did not use a single positive psychology intervention, it is impossible to know whether there is synergy between different interventions. It is also unclear whether there are regional variations in the effectiveness of this positive psychology method, and whether the intervention effect is better in cities with higher education levels.

## Data Availability

The original contributions presented in the study are included in the article/supplementary material. Further inquiries can be directed to the corresponding author.

## References

[1] Giaquinto AN Sung H Newman LA Freedman RA Smith RA Star J . Breast cancer statistics 2024. CA Cancer J Clin. (2024) 74:477–95. doi: 10.3322/caac.21863. PMID: 39352042

[2] Von Au A Dannehl D Dijkstra TMH Gutsfeld R Scholz AS Hassdenteufel K . Breast Cancer and Mental Health: Incidence and Influencing Factors-A Claims Data Analysis from Germany. Cancers (Basel). (2024) 16:3688. doi: 10.3390/cancers16213688. PMID: 39518126 PMC11545012

[3] Seely JM Ellison LF Billette J-M Zhang SX Wilkinson AN . Incidence of Breast Cancer in Younger Women: A Canadian Trend Analysis. Breast (Edinburgh Scotland). (2024) 75:847–854. doi: 10.1177/08465371241246422. PMID: 38664982

[4] Schwieger L Switchenko JM Cao Y Amaniera I Phillips-Reed R Godette K . Intraoperative radiation therapy for early-stage breast cancer. J Surg Oncol. (2024) 130:997–1005. doi: 10.1002/jso.27814. PMID: 39206518

[5] Vasilyeva E Nichol A Bakos B Barton A Goecke M Lam E . Breast conserving surgery combined with radiation therapy offers improved survival over mastectomy in early-stage breast cancer. Am J Surg. (2024) 231:70–3. doi: 10.1016/j.amjsurg.2023.05.005. PMID: 37246127

[6] Wang Y Wang S Tong L Zhuang J Xu Y Wu Y . Relationships between body image, dyadic coping and post-traumatic growth in breast cancer patients: a cross-sectional study. Front Psychol. (2024) 15:1368429. doi: 10.3389/fpsyg.2024.1368429. PMID: 38803834 PMC11129655

[7] Xu W Zhou L Zhao C Zhou Y Chen S Yang L . Evaluating body image disturbance and its influencing factors in breast cancer patients following unilateral mastectomy. Psychiatry Clin Psychopharmacol. (2024) 34:328–35. doi: 10.5152/pcp.2024.24953. PMID: 39772297 PMC11744373

[8] Yigitbas C Bulut A . Exploring psychological help-seeking behaviors and stigma perception among cancer patients: a study on their impact on psychosocial adjustment. Support Care Cancer. (2024) 32:694. doi: 10.1007/s00520-024-08870-z. PMID: 39347823

[9] Wu J Zeng N Wang L Yao L . The stigma in patients with breast cancer: a concept analysis. Asia Pac J Oncol Nurs. (2023) 10:100293. doi: 10.1016/j.apjon.2023.100293. PMID: 37886719 PMC10597826

[10] Zhu H Yang L Yin H Yuan X Gu J Yang Y . The influencing factors of psychosocial adaptation of cancer patients: a systematic review and meta-analysis. Health Serv Insights. (2024) 17:11786329241278814. doi: 10.1177/11786329241278814. PMID: 39291133 PMC11406651

[11] Zhao J Yang DS Liu YQ Wu YK Chen C Li JT . Characteristics of positive and negative effects on the quality of life of breast cancer patients. BMC Psychiatry. (2024) 24:926. doi: 10.1186/s12888-024-06311-z. PMID: 39696113 PMC11657455

[12] Hong W She Z Liu X Yang P . A comparative study of quality of life and psychosocial adaptability following modified radical mastectomy and breast reconstruction. Sci Rep. (2025) 15:45382. doi: 10.1038/s41598-025-29121-z. PMID: 41298903 PMC12748745

[13] Yeoh SA Bowie A Windsor T Russell H Kumar S Beatty L . Effectiveness of positive psychology interventions for cancer survivors: a systematic review and meta-analysis. Cancer Med. (2026) 15:e71368. doi: 10.1002/cam4.71368. PMID: 41499203 PMC12778308

[14] Wang Q Wu Q Wang Y Yang T Chen Q Yang K . The effectiveness of positive psychological intervention based on PERMA model in cancer patients: a systematic review and meta-analysis. J Eval Clin Pract. (2025) 31:e70175. doi: 10.1111/jep.70175. PMID: 40581985

[15] Tian X Zhou X Sun M Yu NX Peng Y Zheng X . The effectiveness of positive psychological interventions for patients with cancer: a systematic review and meta-analysis. J Clin Nurs. (2024) 33:3752–74. doi: 10.1111/jocn.17358. PMID: 38979929

[16] Nisiraiou A Bozas A Kyrou D Stavrogianni K Vasilopoulou M Kalomoiris GM . Post-traumatic growth in adult cancer survivors: a scoping review of psychological factors, predictors, and interventions. Int J Psychol. (2025) 60:e70097. doi: 10.1002/ijop.70097. PMID: 40874359 PMC12392248

[17] Carreiro J Cardoso S Teques P Teques AP Pais-Ribeiro JL . Satisfaction with social support and quality of life among Portuguese patients with breast cancer: mediating effects of coping styles—cross-sectional study. Healthcare. (2025) 13:297. doi: 10.3390/healthcare13030297. PMID: 39942486 PMC11817198

[18] Zhang Y Tang R Wang D Li X Bi L Shi M . Family-centered online positive psychological intervention for breast cancer patients and family caregivers: a single-arm pre-post study of feasibility and preliminary effects. BMC Psychol. (2025) 13:310. doi: 10.1186/s40359-025-02632-0. PMID: 40149009 PMC11951602

[19] Liu S Cai Y Yao S Chai J Jia Y Ge H . Perceived social support mediates cancer and living meaningfully intervention effects on quality of life after breast cancer surgery. Future Oncol. (2024) 20:1675–87. doi: 10.1080/14796694.2024.2370237. PMID: 39011969 PMC11486173

[20] Al-Hendawi M Alodat A Al-Zoubi S Bulut S . A PERMA model approach to well-being: a psychometric properties study. BMC Psychol. (2024) 12:414. doi: 10.1186/s40359-024-01909-0. PMID: 39080800 PMC11290191

[21] Derogatis LR . The psychosocial adjustment to illness scale (PAIS). J Psychosom Res. (1986) 30:77–91. doi: 10.1016/0022-3999(86)90069-3. PMID: 3701670

[22] Gu G Gh F . Response set in the tennessee department of mental health self concept scale. J Clin Psychol. (1965) 21:287–8. doi: 10.1002/1097-4679(196507)21:3<287::aid-jclp2270210323>3.0.co;2-k 14332192

[23] Garthoeffner JL Henry CS Robinson LC . The Modified Interpersonal Relationship Scale: reliability and validity1,2. Psychol Rep. (1993) 73:995–1004. doi: 10.1177/00332941930733pt141

[24] Shuiyuan X . The theoretical basis and research application of social support rating scale. J Clin Psychiatry. (1994) 2:98–100.

[25] Tedeschi RG Calhoun LG . The Posttraumatic Growth Inventory: measuring the positive legacy of trauma. J Trauma Stress. (1996) 9:455–71. doi: 10.1007/BF02103658. PMID: 8827649

[26] Fortin J Rudd É Trudel-Fitzgerald C Cordova MJ Marin M-F Brunet A . Understanding mental health in breast cancer from screening to survivorship: an integrative phasic model and tool. Psychol Health Med. (2025) 30:437–59. doi: 10.1080/13548506.2024.2430796. PMID: 39580147

[27] Arasu K Kou K Goodwin B Chambers S Dunn J Pyke C . Quantifying the influence of psychosocial characteristics, supportive care needs and quality of life on breast cancer survival. Psychooncology. (2025) 34:e70146. doi: 10.1002/pon.70146. PMID: 40215001 PMC11989191

[28] Lu Y Liu Q Dai C Zhai S Geng H Chen C . The impact of patient activation, self-management efficacy, and self-advocacy on symptom burden in breast cancer patients: a longitudinal study. Eur J Oncol Nurs. (2026) 80:103046. doi: 10.1016/j.ejon.2025.103046. PMID: 41338004

[29] Karademas EC Roziner I Simos P Mazzocco K Pat-Horenczyk R Sousa B . Changes over time in self-efficacy to cope with cancer and well-being in women with breast cancer: a cross-cultural study. Psychol Health. (2025) 40:141–54. doi: 10.1080/08870446.2023.2202205. PMID: 37101374

[30] Zhang Y Yan J He H Zhang L Chen L Li N . The trajectories of psychosocial adjustment among young to middle-aged women with breast cancer: a prospective longitudinal study. Eur J Oncol Nurs. (2024) 71:102617. doi: 10.1016/j.ejon.2024.102617. PMID: 38865852

[31] Dibble KE Rosenberg SM Zheng Y Sella T Poorvu P Snow C . Psychosocial and supportive care concerns of young women living with advanced breast cancer: baseline findings from a prospective virtual support intervention study. Support Care Cancer. (2024) 32:336. doi: 10.1007/s00520-024-08557-5. PMID: 38727753

[32] Penberthy JK Stewart AL Centeno CF Penberthy DR . Psychological aspects of breast cancer. Psychiatr Clinics. (2023) 46:551–70. doi: 10.1016/j.psc.2023.04.010. PMID: 37500250

[33] Broadbridge E Greene K Venetis MK Lee LE Banerjee SC Saraiya B . Facilitating psychological adjustment for breast cancer patients through empathic communication and uncertainty reduction. Patient Educ Couns. (2023) 114:107791. doi: 10.1016/j.pec.2023.107791. PMID: 37244129 PMC11046425

[34] Jadidi A Ameri F . Social support and meaning of life in women with breast cancer. Ethiop J Health Sci. (2022) 32:709–14. doi: 10.4314/ejhs.v32i4.6. PMID: 35950056 PMC9341025

[35] Olvera RG Myers SP Gaughan AA Tarver WL Lee S Shiu K . The role of social support in buffering the financial toxicity of breast cancer: a qualitative study of patient experiences. Cancers (Basel). (2025) 17:1712. doi: 10.3390/cancers17101712. PMID: 40427208 PMC12110526

[36] Harmancı P Yıldız E . Associations between psychological resilience and social support with posttraumatic growth in breast cancer patients: a cross-sectional study. Soc Work Public Health. (2024) 39:62–77. doi: 10.1080/19371918.2024.2316876. PMID: 38351648

[37] Huang S Huang M Long F Wang F . Post-traumatic growth experience of breast cancer patients: a qualitative systematic review and meta-synthesis. PloS One. (2025) 20:e0316108. doi: 10.1371/journal.pone.0316108. PMID: 39847563 PMC11756777

